# Electronic cigarettes and health outcomes: epidemiological and public health challenges

**DOI:** 10.1093/ije/dyad059

**Published:** 2023-05-16

**Authors:** Emily Banks, Amelia Yazidjoglou, Grace Joshy

**Affiliations:** National Centre for Epidemiology and Population Health, Australian National University, Canberra, ACT, Australia; National Centre for Epidemiology and Population Health, Australian National University, Canberra, ACT, Australia; National Centre for Epidemiology and Population Health, Australian National University, Canberra, ACT, Australia

## Current evidence about e-cigarettes and health outcomes

Electronic cigarettes (e-cigarettes) are battery-powered devices that heat and aerosolize an ‘e-liquid’. The aerosol is then inhaled by the user. Since their first introduction to broad global markets in 2006–7, use has become increasingly common, particularly among youth.^1^ The population and individual health impacts of e-cigarettes relate to patterns of use, their direct effects on health and their indirect effects, through impacts on smoking behaviour, as well as background disease risks and tobacco control.^2^

Based on the current worldwide evidence summarized in our recent systematic review, use of nicotine e-cigarettes increases the risks of addiction, poisoning, toxicity from inhalation (including seizures) and trauma and burns.^3^ Applying the National Academies of Science, Engineering and Medicine framework for rating the strength of conclusions based on evidence,^4^ the review found strong evidence that, among young non-smokers, uptake of conventional smoking is increased by an average of 3-fold in e-cigarette users versus non-users.^5^ Use of e-cigarettes can cause e-cigarette- or vaping-associated lung injury (EVALI), largely attributable to e-liquids containing tetrahydrocannabinol/vitamin E acetate, although one in eight cases in the largest case series to date were reported to be related to use of nicotine e-cigarettes.^6^

The review found that the effects of e-cigarettes on most important clinical outcomes are not known, due to insufficient or absent evidence. This includes outcomes related to cancer, cardiovascular disease,^7^ respiratory conditions other than lung injury, mental health, development, reproductive health, sleep, neurological conditions other than seizures, and endocrine, olfactory, optical, allergic and haematological conditions.^3^ There was conclusive or substantial evidence that e-cigarettes can cause indoor air pollution, waste and fires.^3^ There was less direct evidence that e-cigarette use can adversely affect cardiovascular health markers, such as blood pressure and heart rate, lung function and adolescent brain development and function.^3^

The review also found limited evidence that freebase nicotine e-cigarettes used in the clinical setting are efficacious smoking cessation aids.^3^ Evidence was also limited that former smokers who use e-cigarettes are around twice as likely to take up smoking again than those not using e-cigarettes.^3^^,^^5^

Based on the systematic review findings, there is currently strong evidence that use of e-cigarettes by non-smokers is harmful to health overall, with multiple health harms and no health benefits identified in this population.^3^ Ex-smokers would be likely to reduce e-cigarette-related health effects if they avoid ongoing e-cigarette use.

Dual tobacco smoking and e-cigarette use is the commonest pattern of e-cigarette use in most settings where evidence is available.^3^^,^^8–10^ The direct health effects of combining smoking and e-cigarette use are unknown.^3^ Dual use may facilitate prolongation of smoking, which would be likely to increase risks.^11^ E-cigarette use in smokers may offset the pressure of many effective tobacco control measures, as evidenced by common reported reasons for use including that, compared with tobacco cigarettes, e-cigarettes are cheaper, more socially acceptable, able to be consumed in settings where tobacco smoking is prohibited and considered less hazardous to health.^3^^,^^8^

Most smokers who quit successfully do so without specific smoking cessation aids.^12^^,^^13^ Therapeutic products must demonstrate an acceptable balance of benefits and risks to be approved by regulatory authorities^14–16^ and a range of approved smoking cessation aids are available.^17^ Internationally, at the time of writing, e-cigarettes are not approved as therapeutic goods. The United States Preventive Services Task Force concluded recently that ‘the current evidence is insufficient to assess the balance of benefits and harms of electronic cigarettes (e-cigarettes) for tobacco cessation in adults, including pregnant persons’ and ‘recommends that clinicians direct patients who use tobacco to other tobacco cessation interventions with proven effectiveness and established safety’.^18^ In England, e-cigarettes combined with behavioural support are recommended as an option for smoking cessation, particularly for smokers who have tried other methods without success,^19^ and in Australia current guidelines list them as an option following use of approved products.^17^ Smoking is extremely harmful and our systematic review findings indicate that the balance of probabilities may be that e-cigarettes benefit smokers who have tried other measures unsuccessfully and who use e-cigarettes to quit tobacco smoking completely and promptly. However, the ultimate balance of safety and efficacy of the use of e-cigarettes for smoking cessation remains unclear; most of their effects on health outcomes are unknown, a number of risks have been identified, evidence on their efficacy for smoking cessation is limited and most smokers using e-cigarettes continue to smoke.^18^

**Figure 1:**
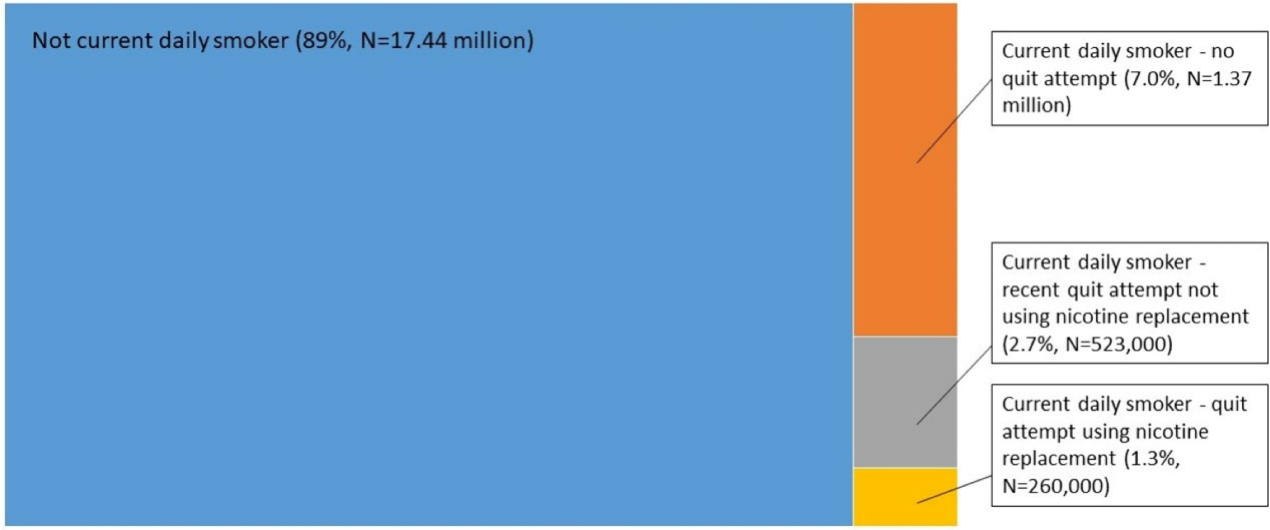
Perspective on contemporary population smoking and quitting patterns in Australia, among people aged 14 and over, including estimated proportions and numbers of smokers making quit attempts and using approved nicotine replacement therapy (data sources: National Drug Strategy Household Survey 2019^8^ and Dono *et al*.^21^)

It is important that evidence regarding e-cigarettes is appropriately contextualized and issues are considered in perspective. This includes noting that in many countries, >80–90% of the population are non-smokers and the main driver of decreasing prevalence of smoking is declining smoking initiation in youth, rather than smoking cessation.^20^ As noted above, smokers seeking medical assistance to quit and using medications are in the minority.^12^^,^^13^ For example, in Australia in 2019, 11% of the population aged 14 and over were current daily smokers (Figure 1).^8^ Among current smokers, recent data indicate 36.3% make at least one quit attempt in a given year and, of these, 33.2% report use of (approved) nicotine replacement therapy.^21^ In Australia, this amounts to around 1.3% of the population aged 14 and over being a current daily smoker who makes a quit attempt using nicotine replacement therapy; an example of a group that could use e-cigarettes. In many countries, potential benefits to smokers—an important minority—are used to justify widespread exposure to e-cigarettes, with risks to young people and the much larger population who are non-smokers.

## Epidemiological and methodological challenges

Evaluation of health impacts focuses primarily on evidence regarding the causal relationship of use of e-cigarettes to health outcomes. In terms of establishing the safety of an exposure, it is also important to consider the extent to which adverse effects can be excluded, which relates to the statistical limits around risk estimates. The context of use is also important. E-cigarette exposure in the general population is increasingly common, which means that moderate elevations in relative risk—around 20% to 30% (i.e. relative risks of 1.2 to 1.3) — would have important implications for public health. Hence, evidence is needed that is capable of both detecting and excluding such elevations in risk. It is therefore necessary to focus on study designs able to provide evidence regarding causality, with sufficient statistical power and quality. Alongside standard issues relating to reliably establishing and excluding exposure-outcome effects, there are some e-cigarette-specific challenges, outlined below.

### Tobacco and e-cigarette industry interests and influence

A major underlying issue in establishing the health impacts of e-cigarettes is the massive ongoing and extremely well resourced efforts of the closely-related and overlapping tobacco and e-cigarette industries^22^^,^^23^ to promote their products and further their interests. These are well documented in the World Health Organization taxonomy of tobacco industry tactics and include efforts to distort and undermine science, influence outcomes and interpretation of findings, influence political and community priorities and divide the tobacco control community.^24^^,^^25^ The e-cigarette industry markets aggressively to children and adolescents, at the same time as claiming that its sole intention is to provide a ‘safer alternative to tobacco’ and to support smokers to quit. Industry funding of scientists and studies provides an important potential source of bias and should be carefully documented and considered when reviewing the evidence.^26^^,^^27^

### Diversity and uncertainty of e-cigarette exposure and rapid change

The health effects of e-cigarettes relate chiefly to: (i) the chemicals emitted by the e-cigarette, which originate from the e-liquid, chemical reactions in the heating element and the device itself^28^; (ii) the device; and (iii) aspects impacting on smoking behaviour. There may also be summative and/or synergistic effects of the combination of these exposures.

E-liquids generally contain chemical solvents propylene glycol and vegetable glycerine, and the vast bulk of e-cigarette use is of those delivering nicotine^29^; these commonalities underpin investigation of health effects. Bearing this in mind, there are many thousands of e-cigarette devices and e-liquids, with >17 000 flavours available currently and hundreds being introduced to the market on a monthly basis. Hence, e-cigarette use in the population results in exposure to many thousands of different chemical combinations, and varying doses of these chemicals.^28^

Certain health effects may relate to specific chemical constituents or device properties, and risk may therefore vary between e-cigarettes. Currently, a major division is between use of freebase nicotine and nicotine salt products. Nicotine salt preparations make high concentrations of nicotine more palatable by reducing throat irritation, and are the main form of nicotine used in disposable and pod e-cigarettes.^28^ These e-cigarette types are small and easily concealed and are heavily marketed to children and adolescents, including through their design. They have been identified as key drivers of widespread youth use of e-cigarettes, including in Canada and the USA.^3^^,^^30^ Many commonly used disposable nicotine salt products deliver high concentrations of nicotine, which would increase the risk of nicotine toxicity via inhalation, including seizures. Evidence also suggests that nicotine salt products confer higher risks of dependency than other types.^31^ EVALI has largely—but not exclusively—been attributed to e-liquids containing tetrahydrocannabinol/vitamin E acetate. ‘Popcorn lung’ is bronchiolitis obliterans attributed to inhalation of diacetyl used as a flavouring in microwave popcorn; concerns have been raised about use of diacetyl in e-liquids.^32^^,^^33^ Users of ‘open’ e-cigarettes fill a reservoir in the device with the e-liquid. Such e-liquids are either premixed or involve the mixing of the different components, often including the dilution of more concentrated liquid nicotine. Concentrated liquid nicotine is highly toxic and at-home dilution of high-concentration nicotine increases the risk of accidental and intentional poisoning, including in children.^3^ It also increases the risk of e-liquid adulteration. The risk of injuries related to exploding batteries varies according to the construction of the device and the quality of the batteries within it.

It is often difficult to know with accuracy what the components of an e-liquid are, as labelling is variable and often absent or inaccurate. Many studies do not report on the types of e-cigarettes used by participants and/or many participants are unable to report on the specific type of e-cigarette or combinations of e-cigarettes that they have used. There can also be issues generalizing between e-liquid types, and rapid change creates a ‘moving target’ for monitoring and research. For example although much contemporary use is of nicotine salt products,^34^ most of the studies to date, including randomized trials of smoking cessation and observational studies of smoking uptake, are of e-cigarettes delivering freebase nicotine. A major related factor influencing e-cigarette exposure and risks is user behaviour: use confined to smoking cessation would lead to very different population risks compared with use primarily among youth who are not established smokers.^35^

### Limited data and the relatively recent introduction of e-cigarettes as a population exposure

Population exposure to e-cigarette use has only become substantial since around 2010. Certain immediate or short-term outcomes are already discernible, particularly those where cause and effect are able to be ascertained at an individual level and where appropriate surveillance systems are in place. For example, the addictive effects of e-cigarettes are well established, as is their ability to cause poisoning, trauma, burns and toxicity through inhalation. The EVALI outbreak was detected and addressed rapidly. Effects on the uptake of smoking in young people have also been able to be assessed fairly quickly, with >25 studies available in our recent meta-analysis.^5^ For some e-cigarette components and e-liquid constituents, toxicological and other data on their impacts is informative—for example, many effects of nicotine are well established.

It will be some time before it is possible to reliably ascertain many important effects of e-cigarettes on health, due to the time needed to conduct research and because some conditions take many years to develop. This, along with the other issues outlined in this article, means that evidence on major clinical conditions such as cancer, cardiovascular disease, mental illness, respiratory conditions other than EVALI and adverse reproductive outcomes is very limited.

### The need to focus on high-quality evidence, including clinical endpoints rather than intermediate markers

Evidence should focus on clinically meaningful health outcomes including disease endpoints such as invasive cancer, cardiovascular disease events (e.g. myocardial infarction and stroke), respiratory diseases (e.g. asthma, infections and chronic obstructive pulmonary disease) and addiction and dependency-related outcomes. In the absence of this type of evidence, it is tempting to focus on biomarkers, so-called ‘intermediate’ outcomes (such as arterial wall thickness) and pathophysiological parameters (e.g. heart rate, blood pressure). Whereas these may be informative, they are not reliable substitutes for clinical outcomes and there are many examples of the risks relating to relying on this type of evidence for making decisions on safety.

### Choice of comparator

In epidemiological studies, a comparator has two major related but distinct functions: (i) as a reference group supporting reliable quantification of the effect of an exposure; and (ii) as a counterfactual in understanding the likely impact of an exposure. For e-cigarettes, the comparator needs to factor in cigarette smoking status, for reasons detailed further in the section below.

The most appropriate reference group for analyses quantifying the distinct health effects and safety of e-cigarettes is people who have neither used e-cigarettes nor smoked.^3^ Inclusion of people who smoke in the reference group causes major problems quantifying direct e-cigarette effects (see below). Neither smoking nor using e-cigarettes is the most appropriate counterfactual to using e-cigarettes for the considerable majority of the population who are non-smokers in virtually all countries, particularly for young people (Figure 1).

Very few exposures are as harmful as smoking, and quitting tobacco smoking is highly beneficial to health, compared with the counterfactual of continuing to smoke. For people who currently smoke, multiple e-cigarette-related counterfactuals apply. For ongoing dual smokers/e-cigarette users, there is the comparison with people who continue to smoke and who do not use e-cigarettes. Since it is unclear whether smokers who use e-cigarettes may prolong smoking, the counterfactual of quitting by means other than e-cigarettes may also apply. For ex-smokers who have used e-cigarettes to quit completely, there are counterfactuals of continuing to smoke and of quitting smoking by other means.

There are active efforts to shift attention to smoking as the comparator for any use of e-cigarettes—that is, use by smokers and non-smokers, and use for smoking cessation and for other purposes. This is a tactic of the tobacco and e-cigarette industry, typified by use of statements along the lines that e-cigarettes are ‘safer than smoking’ for the broad marketing of these products, including to non-smokers. Comparisons with smoking are also implicit in the framing of e-cigarettes as a ‘harm reduction’ measure. The exceptional harm of tobacco smoking means that such comparisons are highly advantageous to industry, and cannot be used as evidence of safety in absolute terms. E-cigarette effects on disease risk in smokers remain important, but they must be recognized as distinct from total population impacts and impacts in non-smokers. In particular, comparisons of e-cigarettes with smoking should be avoided in relation to use in non-smokers (the comparison should be with breathing air), potential harm reduction in one population should not automatically be considered harm reduction at a population level and the World Health Organization is clear that use of alternative tobacco products—such as e-cigarettes—in non-smokers cannot be considered harm reduction.^36^

### Bias and confounding, particularly in relation to tobacco smoking

Quantifying the health impacts of e-cigarettes requires differentiation of their likely effects from those of other factors that use is related to. Potential confounding factors include age, sex, socioeconomic status, ethnicity/race, adiposity, physical activity, reproductive factors, alcohol consumption, other drug use, environmental tobacco smoke and pre-existing physical and mental health conditions, as well as particular issues in relation to combustible tobacco smoking.

Smoking markedly increases the risk of a very wide range of health conditions.^37^^,^^38^ Contemporary data from Australia demonstrate that lung cancer incidence in current smokers is 18 times that of never-smokers, with a 6-fold risk in former smokers.^39^ Cardiovascular disease risk in current versus never-smokers is 2–3-fold^40^ and the risk of dying of chronic obstructive pulmonary disease is more than 30-fold.^37^ In addition, risk increases with increasing duration of smoking and there are large elevations in risk with increasing number of cigarettes smoked per day. For example, compared with never-smokers, the hazard ratio for lung cancer incidence is 9.22 [95% confidence interval (CI) 5.14–16.55] for current smokers of 1–5 cigarettes per day and 38.6 (95% CI 25.7–58.1) with >35 cigarettes per day.^39^ Risks also vary according to age and background risk of disease and among ex-smokers, disease risk diminishes with time since quitting.^37^^,^^41^ The tendency of current smokers to quit when they become ill (the ‘sick quitter’ effect) is well established and adds further complexity to quantifying smoking effects.^42^

In the situation where an exposure is related to smoking behaviour and smoking has a strong relationship to the outcome, reliable quantification of exposure-outcome relationships independent of smoking is difficult if not impossible, if smokers are included in the analyses. Adjustment of analyses for smoking generally can only account for relatively broad categories of current, past and never smoking, with limited ability to account for fine categories of intensity and other smoking attributes, leading to major issues with residual confounding.^43^ Hence the most—and often the only—reliable evidence comes from confining analyses to people who have never smoked. Examples of where this method is used include studies of the relationship of lung cancer to environmental tobacco smoke^44^^,^^45^ and risk factors other than smoking.^46^

E-cigarette use relates strongly to smoking.^5^^,^^47–49^ E-cigarette use is more common in current or ex-smokers, compared with never-smokers, and concurrent (dual) use of both e-cigarettes and tobacco cigarettes is a frequent pattern of use in many places.^9^^,^^10^^,^^50^ There are complex differences in tobacco smoking behaviour between dual users and exclusive tobacco smokers, including in tobacco smoking intensity and duration, as well as other factors. In addition, smokers who become ill may take up e-cigarette use, aiming to reduce or quit smoking (sometimes termed ‘sick switching’). These issues are likely to impact on effect estimates when comparing ex-smokers who use e-cigarettes with those who do not; similar problems will occur when comparing dual users with exclusive cigarette smokers. If the reference group is never-smokers, then comparisons with e-cigarette users who are current or ex-smokers are particularly problematic.

Establishing the safety of e-cigarettes requires studies able to detect and exclude relative risk increases from e-cigarette exposure of 20–30%. However, this magnitude of elevation in disease risk is small in comparison with that resulting from relatively minor differences in the number of cigarettes smoked per day among smokers—particularly for diseases such as lung cancer and chronic obstructive pulmonary disease. Hence for many conditions, residual confounding with tobacco smoking is likely to overwhelm the ability to reliably quantify—and exclude—direct effects of e-cigarettes, even following adjustment. This means that never-smokers who use e-cigarettes and who do not go on to smoke, are the most reliable exposed population in which to quantify the direct health effects of e-cigarette use.^4^^,^^51^ The fact that some never-smokers who initiate e-cigarette use ultimately start smoking further limits evidence about health outcomes attributable to e-cigarette use.^4^^,^^51^

### Effect modification/statistical interaction

Exposure-outcome relative risks tend to differ in magnitude between subgroups which vary in absolute rates of disease. For example, cardiovascular disease mortality rates vary by age. Reductions in cardiovascular mortality are observed with blood pressure-lowering treatments in all age groups and the magnitude of this effect varies with age; greater relative risk reductions are observed in younger age groups and greater absolute risk reductions are observed in older age groups.^52^

Rates of disease differ markedly between current, past and never-smokers.^37^ Even if it was possible to reliably ascertain relative risks in populations that included smokers, the relative effect of e-cigarettes would be likely to differ between them. Moreover, the combined effects of smoking and e-cigarettes may differ from their separate effects. This highlights the importance of conducting stratified analyses, including quantifying the effects of e-cigarettes separately in people who currently smoke, ex-smokers and never-smokers, bearing in mind the issues with confounding and bias outlined above.

### Statistical power

Broadly speaking, reliable quantification of an exposure-outcome relationship requires sufficient numbers of outcome events among the exposed and unexposed to estimate the parameters of interest with required precision, taking account of matters such as measurement error, confounding and bias. All of the issues noted earlier in the paper have a bearing on statistical power.

In addition, whereas certain important health outcomes, such as addiction, mental health issues and asthma occur across the lifespan, most of the major clinical disease outcomes of interest in examining the effects of e-cigarettes—such as cancer, cardiovascular disease and chronic obstructive pulmonary disease—largely occur at older ages. Use of e-cigarettes at older ages (e.g. those aged >40 years) is almost always among people who currently smoke or have smoked in the past; there is very little use among older never-smokers.^8^ E-cigarette use among never-smokers and people who are not established smokers is a common pattern in youth and, since smoking habits are generally not established until people are in their mid-20s, use below this age is chiefly not aimed at smoking cessation.

Hence, at the age where the vast majority of disease events occur, use of e-cigarettes is almost exclusively in smokers, making it very difficult to separate the effects of e-cigarettes from those of variations in smoking behaviour (see above). At ages where e-cigarette use among never-smokers is more common, chronic disease events—apart from those mentioned above—are generally rare. For example, in a major large cohort study of e-cigarettes and respiratory outcomes, 99.4% of e-cigarette users were either current or former smokers.^43^

Even in large studies, the stratification necessary to be assured of effects independent of smoking may leave limited power to detect effects in never-smokers, particularly as disease events will tend to occur disproportionately in people who smoke.^53^

## Implications

Despite the methodological and other issues identified, evidence regarding the health impacts of e-cigarettes is accumulating rapidly. The risks that have been identified are sufficient to conclude that use in non-smokers, particularly youth, is harmful to health. For most major health outcomes, the effects of e-cigarettes are uncertain.

A key ongoing challenge for epidemiology and public health is not only the obtaining of reliable evidence on e-cigarettes, but also its interpretation and use as well as its misuse. Effective public health action requires meticulous consideration of evidence, the assessment of risks and benefits overall and to multiple population groups and effective measures to manage risk. Like aviation and surgery, it is not about ‘taking a punt’ on something that seems intuitively appealing or hoping that certain measures will work. In particular, it is not about assuming that scaling up something that appears to work for an individual or smaller group would automatically be beneficial for the population as a whole. Mistakes in public health cost millions of lives and span generations. Public health also requires active opposition to forces—such as the tobacco industry—which seek to profit at the expense of the health and wellbeing of the community.

Public health action on e-cigarettes, including regulatory decision making, must manage their identified risks as well as uncertainty. The precautionary principle highlights the importance of avoiding exposure to an agent when there is uncertainty combined with scientifically plausible evidence of serious risks to human health, especially those affecting future generations.^54^ It clarifies that the onus is on proving safety—including by the proponents of an intervention—before allowing widespread exposure, and emphasizes the need to take action to prevent harm, rather than solely reacting once harm is done.^55^ Governments are important stakeholders and regulation of e-cigarettes varies widely internationally, with at least 32 countries banning them totally, 79 having regulations that do not include a total ban and 84 having no regulations in place, at the time of writing.^56^ Overall, the evidence is supportive of efforts in many jurisdictions to avoid e-cigarette use in the general population, particularly in non-smokers and youth. It is also supportive of the World Health Organization position that non-pregnant smokers who switch completely and promptly to an appropriately regulated [preferably medically approved] e-cigarette may benefit, bearing in mind the lack of evidence on long-term impacts (brackets ours).^56^

It is important that evidence regarding the health impacts of e-cigarettes is interpreted and applied independent of industry influence. Industry profits from maximizing sales and hence promotes widespread e-cigarette use, and research to understand and efforts to control e-cigarette use are hampered by its activities. Industry actively seeks to conflate evidence of potential efficacy for smoking cessation in some individuals, groups or settings, with the idea that such use is beneficial overall in smokers and that widespread e-cigarette use would improve public health. If the tobacco and e-cigarette industry would ‘stay in its box’ and truly only target smokers who want to use their products to quit, things would be very different. It is worth conducting a thought experiment to consider what this would look like: no marketing to children and adolescents; no flavours designed to appeal to children (such as bubble gum and candy floss); use primarily for smoking cessation; and submission of e-cigarette products for regulatory approval for smoking cessation.

##  

The issues outlined in this article highlight the importance of investment in high-quality independent research to address outstanding and ongoing issues regarding e-cigarettes, including but not limited to: data to effectively monitor e-cigarette and tobacco product use, including tobacco smoking, particularly among youth; studies of health impacts, able to separate e-cigarette and smoking effects; evidence informing the balance of quality, safety and efficacy of e-cigarettes as aids for smoking cessation; research on countering tobacco and e-cigarette industry influence; and research on effective measures to manage identified risks related to e-cigarettes, including avoiding e-cigarette use in non-smokers, especially youth. Evidence on how to effectively control inappropriate marketing to children and youth, particularly via social media, is a priority.^22^ It is also important to acknowledge uncertainty and to resist the temptation to draw inappropriately on substandard evidence, in circumstances where high-quality evidence is lacking. Research should make the most of policy heterogeneity in the approach to e-cigarettes, to inform effective regulatory responses to maximize the health of current and future generations, including through accelerated tobacco control.

## Ethics approval

No ethical approval was required as this paper is based on publicly available data.

## Data Availability

This paper is based on publicly available data.

## References

[1] Yoong SL , Hall A, Leonard A et al Prevalence of electronic nicotine delivery systems and electronic non-nicotine delivery systems in children and adolescents: a systematic review and meta-analysis. Lancet Public Health 2021;6:e661–73. 34274048 10.1016/S2468-2667(21)00106-7 PMC8390387

[2] Banks E , Martin M, Harris M. Framework for the public health assessment of electronic cigarettes. Tob Control 2022;31:608–14. 33958423 10.1136/tobaccocontrol-2020-056271

[3] Banks E , Yazidjoglou A, Brown S et al Electronic Cigarettes and Health Outcomes: Systematic Review of Global Evidence. Canberra: National Centre for Epidemiology and Population Health, 2022.

[4] National Academies of Sciences, Engineering, and Medicine. *Public Health Consequences of e-Cigarettes.* Report No.: 0309468345. Washington, DC: National Academies Press, 2018. 10.17226/24952 (6 December 2022, date last accessed). 29894118

[5] Baenziger O , Ford L, Yazidjoglou A, Joshy G, Banks E. E-cigarette use and combustible tobacco cigarette smoking uptake among non-smokers, including relapse in former smokers: umbrella review, systematic review and meta-analysis. BMJ Open 2021;11:e045603. 10.1136/bmjopen-2020-045603 PMC8011717 33785493

[6] Krishnasamy VP , Hallowell BD, Ko JY et al; Lung Injury Response Epidemiology/Surveillance Task Force. Update: characteristics of a nationwide outbreak of e-cigarette, or vaping, product use-associated lung injury: United States, August 2019-January 2020. MMWR Morb Mortal Wkly Rep 2020;69:90–94. 31971931 10.15585/mmwr.mm6903e2 PMC7367698

[7] Wold LE , Tarran R, Alexander LEC et al; American Heart Association Council on Basic Cardiovascular Sciences; Council on Arteriosclerosis, Thrombosis and Vascular Biology; Council on Hypertension; and Stroke Council. Cardiopulmonary consequences of vaping in adolescents: a scientific statement from the American Heart Association. Circ Res 2022;131:e70–82. 35726609 10.1161/RES.0000000000000544

[8] Australian Institute of Health and Welfare. *National Drug Strategy Household Survey 2019, Tobacco Smoking-Supplementary Tables*. Canberra: Australian Government, 2020. 10.25816/e42p-a447 (6 December 2022, date last accessed).

[9] Reid JL , Rynard VL, Czoli CD, Hammond D. Who is using e-cigarettes in Canada? Nationally representative data on the prevalence of e-cigarette use among Canadians. Prev Med 2015;81:180–83. 26348453 10.1016/j.ypmed.2015.08.019

[10] Sung H-Y , Wang Y, Yao T, Lightwood J, Max W. Polytobacco use and nicotine dependence symptoms among US adults, 2012–2014. Nicotine Tob Res 2018;20:S88–98. 30125019 10.1093/ntr/nty050 PMC6093419

[11] WHO Study Group on Tobacco Regulation. Report on the Scientific Basis of Tobacco Product Regulation: eighth Report of a WHO Study Group. Geneva: World Health Organization, 2021.

[12] Chapman S , MacKenzie R. The global research neglect of unassisted smoking cessation: causes and consequences. PLoS Med 2010;7:e1000216. 20161722 10.1371/journal.pmed.1000216 PMC2817714

[13] Soulakova JN , Crockett LJ. Unassisted quitting and smoking cessation methods used in the United States: analyses of 2010–2011 tobacco use supplement to the current population survey data. Nicotine Tob Res 2016;20:30–39. 10.1093/ntr/ntw273 PMC5896463 27798084

[14] European Medicines Agency. *What We Do*. 2022. https://www.ema.europa.eu/en/about-us/what-we-do (6 December 2022, date last accessed).

[15] Food and Drug Administration. *What We Do: The Food and Drug Administration*. 2018. https://www.fda.gov/about-fda/what-we-do#:~:text=FDA%20Basics-,FDA%20Mission,and%20products%20that%20emit%20radiation (6 December 2022, date last accessed).

[16] Therapeutic Goods Administration. *Product Regulation According to Risk: Therapeutic Goods Administration.* https://www.tga.gov.au/product-regulation-according-risk (6 December 2022, date last accessed).

[17] Royal Australian College of General Practitioners. *Supporting Smoking Cessation: A Guide for Health Professionals: Pharmacotherapy for Smoking Cessation*. 2021. https://www.racgp.org.au/clinical-resources/clinical-guidelines/key-racgp-guidelines/view-all-racgp-guidelines/supporting-smoking-cessation/pharmacotherapy-for-smoking-cessation (6 December 2022, date last accessed).

[18] US Preventive Services Task Force. Interventions for Tobacco Smoking Cessation in Adults, Including Pregnant Persons: US Preventive Services Task Force recommendation statement. JAMA 2021;325:265–79. 33464343 10.1001/jama.2020.25019

[19] McNeill A , Brose L, Calder R, Simonavicius E, Robson D. *Vaping in England: An Evidence Update Including Vaping for Smoking Cessation, February 2021. A Report Commissioned by Public Health England*. London: Public Health England, 2021.

[20] Australian Institute of Health Welfare. *Tobacco*. Canberra: Australian Institute of Health and Welfare, 2022. https://www.aihw.gov.au/reports/alcohol/alcohol-tobacco-other-drugs-australia/contents/drug-types/tobacco (6 December 2022, date last accessed).

[21] Dono J , Martin K, Bowden J, Miller C. A population-level analysis of changes in Australian smokers’ preferences for smoking cessation support over two decades: from 1998 to 2017. Lancet Reg Health West Pac 2022;19:100342. 35024667 10.1016/j.lanwpc.2021.100342 PMC8669336

[22] Tobacco Control Research Group. *E-cigarettes: University of Bath*. 2022. https://tobaccotactics.org/wiki/e-cigarettes/ (6 December 2022, date last accessed).

[23] Greenhalgh E , Scollo M. InDepth 18B: electronic cigarettes (e-cigarettes). In: *Tobacco in Australia: Facts and Issues*. Melbourne, SA: Cancer Council Victoria, 2019. https://www.tobaccoinaustralia.org.au/chapter-18-harm-reduction/indepth-18b-e-cigarettes/18b-1-the-ecigarettemarket (29 July 2022, date last accessed).

[24] World Health Organization. Tobacco Industry Interference with Tobacco Control. Geneva: WHO Press, 2008. https://www.who.int/publications/i/item/9789241597340 (6 December 2022, date last accessed).

[25] World Health Organization. *Technical Resource for Country Implementation of WHO Framework Convention on Tobacco Control Article 5.3 on the Protection of Public Health Policies with Respect to Tobacco Control from Commercial and Other Vested Interests of the Tobacco Industry*. Geneva: World Health Organization, 2012. https://www.who.int/publications/i/item/9789241503730 (6 December 2022, date last accessed).

[26] Pisinger C, Godtfredsen N, Bender AM. A conflict of interest is strongly associated with tobacco industry–favourable results, indicating no harm of e-cigarettes. *Prev Med* 2019;199:124–131. 10.1016/j.ypmed.2018.12.011 30576685

[27] Lundh A , Lexchin J, Mintzes B, Schroll JB, Bero L. Industry sponsorship and research outcome. Cochrane Database Syst Rev 2017;2:Mr000033. 28207928 10.1002/14651858.MR000033.pub3 PMC8132492

[28] SCHEER (Scientific Committee on Health, Environmental and Emerging Risks).Scientific Opinion on Electronic Cigarettes. Luxembourg: European Commission, 2021. https://health.ec.europa.eu/system/files/2022-08/scheer_o_017.pdf (6 December 2022, date last accessed).

[29] Marynak KL , Gammon DG, Rogers T, Coats EM, Singh T, King BA. Sales of nicotine-containing electronic cigarette products: United States, 2015. Am J Public Health 2017;107:702–05. 28323467 10.2105/AJPH.2017.303660 PMC5388940

[30] Government of Canada. *Vaping Products: New Limits on Nicotine Concentration and Consultation on Flavour Restrictions*. 2021. https://www.canada.ca/en/health-canada/news/2021/06/backgrounder-vaping-products--new-limits-on-nicotine-concentration-and-consultation-on-flavour-restrictions.html (29 July 2022, date last accessed).

[31] Lee SJ , Rees VW, Yossefy N, Emmons KM, Tan ASL. Youth and young adult use of pod-based electronic cigarettes from 2015 to 2019: a systematic review. JAMA Pediatr 2020;174:714–20. 32478809 10.1001/jamapediatrics.2020.0259 PMC7863733

[32] Allen JG , Flanigan SS, LeBlanc M et al Flavoring chemicals in e-cigarettes: diacetyl, 2,3-pentanedione, and acetoin in a sample of 51 products, including fruit-, candy-, and cocktail-flavored e-cigarettes. Environ Health Perspect 2016;124:733–39. 26642857 10.1289/ehp.1510185 PMC4892929

[33] Eshraghian EA , Al-Delaimy WK. A review of constituents identified in e-cigarette liquids and aerosols. Tob Prev Cessat 2021;7:10. 33585727 10.18332/tpc/131111 PMC7873740

[34] Government of Canada. *Nicotine Concentration in Vaping Products Regulations: SOR/2021–123; P. C. 2021–518 10 June 2021*. Part II, Vol. 155, Number 13. Ottawa: Canada Gazette, 2021. https://gazette.gc.ca/rp-pr/p2/2021/2021-06-23/html/sor-dors123-eng.html (6 December 2022, date last accessed).

[35] Al-Delaimy WK , Sim F. Electronic cigarettes and public health: a policy brief. Int J Epidemiol 2021;50:705–10. 33615371 10.1093/ije/dyab017

[36] WHO Scientific Advisory Committee on Tobacco Product Regulation, WHO Tobacco Free Initiative. *SACTob Statement of Principles Guiding the Evaluation of New or Modified Tobacco Products /Scientific Advisory Committee on Tobacco Product Regulation (SACTob)*. Report No.: 9241590513 (Eng). Geneva: World Health Organization, 2003. https://www.who.int/publications/i/item/statement-of-principles-guiding-the-evaluation-of-new-or-modified-tobacco-products (6 December 2022, date last accessed).

[37] Pirie K , Peto R, Reeves GK, Green J, Beral V Million Women Study Collaborators The 21st century hazards of smoking and benefits of stopping: a prospective study of one million women in the UK. Lancet 2013;381:133–41. 23107252 10.1016/S0140-6736(12)61720-6 PMC3547248

[38] U.S. Department of Health and Human Services. *The Health Consequences of Smoking: 50 Years of Progress. A Report of the Surgeon General.* Atlanta, GA: U.S. Department of Health and Human Services, Centers for Disease Control and Prevention, National Center for Chronic Disease Prevention, Health Promotion, Office on Smoking and Health, 2014. https://www.ncbi.nlm.nih.gov/books/NBK179276/ (6 December 2022, date last accessed).

[39] Weber MF , Sarich PEA, Vaneckova P et al Cancer incidence and cancer death in relation to tobacco smoking in a population-based Australian cohort study. Int J Cancer 2021;149:1076–88. 34015143 10.1002/ijc.33685

[40] Banks E , Joshy G, Korda RJ et al Tobacco smoking and risk of 36 cardiovascular disease subtypes: fatal and non-fatal outcomes in a large prospective Australian study. BMC Med 2019;17:128. 31266500 10.1186/s12916-019-1351-4 PMC6607519

[41] Banks E , Joshy G, Weber MF et al Tobacco smoking and all-cause mortality in a large Australian cohort study: findings from a mature epidemic with current low smoking prevalence. BMC Med 2015;13:38. 25857449 10.1186/s12916-015-0281-z PMC4339244

[42] Langhammer A , Johnsen R, Holmen J, Gulsvik A, Bjermer L. Cigarette smoking gives more respiratory symptoms among women than among men. The Nord-Trondelag Health Study (HUNT). J Epidemiol Community Health 2000;54:917–22. 11076988 10.1136/jech.54.12.917 PMC1731608

[43] Bhatta DN , Glantz SA. Association of e-cigarette use with respiratory disease among adults: a longitudinal analysis. Am J Prev Med 2020;58:182–90. 31859175 10.1016/j.amepre.2019.07.028 PMC6981012

[44] Vineis P , Hoek G, Krzyzanowski M et al Lung cancers attributable to environmental tobacco smoke and air pollution in non-smokers in different European countries: a prospective study. Environ Health 2007;6:7. 17302981 10.1186/1476-069X-6-7 PMC1803768

[45] Zhong L , Goldberg MS, Parent ME, Hanley JA. Exposure to environmental tobacco smoke and the risk of lung cancer: a meta-analysis. Lung Cancer 2000;27:3–18. 10672779 10.1016/s0169-5002(99)00093-8

[46] Pirie K , Peto R, Green J, Reeves GK, Beral V; Million Women Study Collaborators. Lung cancer in never smokers in the UK Million Women Study. Int J Cancer 2016;139:347–54. 26954623 10.1002/ijc.30084 PMC4864444

[47] Adermark L , Galanti MR, Ryk C, Gilljam H, Hedman L. Prospective association between use of electronic cigarettes and use of conventional cigarettes: a systematic review and meta-analysis. ERJ Open Res 2021;7:00976–2020. 34262971 10.1183/23120541.00976-2020 PMC8273394

[48] O'Brien D , Long J, Quigley J, Lee C, McCarthy A, Kavanagh P. Association between electronic cigarette use and tobacco cigarette smoking initiation in adolescents: a systematic review and meta-analysis. BMC Public Health 2021;21:954. 34078351 10.1186/s12889-021-10935-1 PMC8173887

[49] Owusu D , Huang J, Weaver SR et al Patterns and trends of dual use of e-cigarettes and cigarettes among U.S. adults, 2015–2018. Prev Med Rep 2019;16:101009. 31763161 10.1016/j.pmedr.2019.101009 PMC6861646

[50] Cornelius ME , Wang TW, Jamal A, Loretan CG, Lj N. Tobacco product use among adults—United States, 2019. MMWR Morb Mortal Wkly Rep 2020;69:1736–42. 33211681 10.15585/mmwr.mm6946a4 PMC7676638

[51] Byrne S , Brindal E, Williams G. E-Cigarettes, Smoking and Health. A Literature Review Update. Canberra: CSIRO, 2018.

[52] Rahimi K , Bidel Z, Nazarzadeh M et al Age-stratified and blood-pressure-stratified effects of blood-pressure-lowering pharmacotherapy for the prevention of cardiovascular disease and death: an individual participant-level data meta-analysis. Lancet 2021;398:1053–64. 34461040 10.1016/S0140-6736(21)01921-8 PMC8473559

[53] Freedman N , Abnet CC, Leitzmann MF et al A prospective study of tobacco, alcohol, and the risk of esophageal and gastric cancer subtypes. Am J Epidemiol 2007;165:1424–33. 17420181 10.1093/aje/kwm051

[54] World Commission on the Ethics of Scientific Knowledge and Technology. *The Precautionary Principle*. Paris: United Nations Educational Scientific and Cultural Organization, 2005. https://unesdoc.unesco.org/ark:/48223/pf0000139578 (6 December 2022, date last accessed).

[55] Pearce N , Public health and the precautionary principle. In: Martuzzi M, Tickner JA, eds. The Precautionary Principle. Protecting Public Health, the Environment and the Future of Our Children. Geneva: World Health Organization, Regional Office for Europe, 2004, pp.49–62.

[56] World Health Organization. *WHO Report on the Global Tobacco Epidemic 2021: Addressing New and Emerging Products*. Geneva: World Health Organization, 2021. https://www.who.int/publications/i/item/9789240032095 (6 December 2022, date last accessed).

